# Causation vs. causal explanation: a response to Axmacher

**DOI:** 10.3389/fpsyg.2014.01148

**Published:** 2014-10-13

**Authors:** Elisa Galgut

**Affiliations:** Philosophy, University of Cape TownCape Town, South Africa

**Keywords:** epistemology, philosophy of psychoanalysis, neuropsychoanalysis, causal explanation, hermeneutic explanations

In his paper, “Causation in psychoanalysis,” Dr Nikolai Axmacher raises and responds to three arguments that claim that psychoanalytic explanations and causal explanations in the neurological sciences are mutually inconsistent. These arguments, he claims, are raised by many opponents of neuropsychoanalysis, who argue that psychoanalytic explanations, because they are hermeneutic in character, cannot be consistent with causal explanations in the sciences. Axmacher disputes these arguments, and attempts to show that the apparent differences between hermeneutical and causal explanations are *merely* apparent; he thereby hopes to defend “the neuropsychoanalytic endeavor” (Axmacher, 2013, p. 3). I examine Axmacher's responses to the three arguments he raises; I shall argue that two of them are indeed insufficient to raise concerns about the neuropsychoanalytic project, but one of the arguments—Argument Two—does raise concerns that Axmacher's responses do not consider. Axmacher hopes to show that causal and hermeneutic explanations are, at least in principle, consistent with each other by showing that hermeneutic psychoanalytic explanations also appeal to causal principles. I argue that even if the latter claim is true, fundamental differences between psychoanalytic explanations, and scientific causal explanations, remain.

At the heart of the inconsistency between psychoanalysis and neuroscience is, claims Axmacher, a difference in kinds of explanation: psychoanalysis relies on hermeneutic explanations, which “are epistemologically problematic because they typically act in a retroactive manner … but do not make predictions” (Axmacher, 2013, p. 1), while neuroscience appeals to causal explanations that both explain and predict on the basis of scientific laws. Axmacher examines three specific arguments that “suggest that hermeneutic reconstructions are fundamentally inconsistent with causal explanations” (Axmacher, 2013, p. 2) in the hopes of refuting them. Argument One—the “Freedom and Causality” argument—is based on Habermas's understanding of the nature of psycho-therapeutic work in lifting repression. Habermas conceptualizes repression in the unconscious in terms of causal processes, which are “suppressive” and “dominating,” whereas psychoanalysis is a hermeneutic process that is “conceptualized as a communicative process governed by the idea of the ‘unforced force of the better argument’” (Axmacher, 2013, p. 2). Argument Two claims that “causal explanations state an inevitable and lawful relationship between cause and effect” (Axmacher, 2013, p. 2), while “hermeneutic explanations rely on deferred reconstructions” (Axmacher, 2013, p. 2). What is meant by “deferred reconstructions” is not spelled out, but I take it to mean that hermeneutic explanations do not refer to deterministic causal laws. Argument Three claims that the relationship between a cause and its effect in empirical observations needs to be discovered and is falsifiable, while hermeneutic-type explanations, which are typically given in the first-person, are both unsurprising and unfalsifiable, and hence not causal.

I first examine Axmacher's responses to arguments One and Three, leaving discussion about Argument Two for later. Against Argument One, Axmacher rightly raises a concern that Habermas's account of repression—which Habermas describes as being governed by causal laws characteristic of the natural sciences—is inconsistent with his understanding of the hermeneutic nature of psychoanalysis more broadly. Axmacher objects that “it remains unclear how the therapeutic transition from causal laws to hermeneutic relationships should occur” (Axmacher, 2013, p. 2). This seems an appropriate objection to Habermas, whose understanding of repression conflicts with the way that many psychoanalysts understand it. Freud, for example, writes about repression in psychological terms: “*the essence of repression lies simply in turning something away, and keeping it at a distance, from the conscious”* [Freud, 1985 (1915), p. 147, italics in original]. This description is clearly not a complete analysis of the nature of repression, but there can be no doubt that Freud, despite being a materialist, explains repression in psychological, not scientific, terms. I thus agree with Axmacher's concerns regarding Habermas's characterization of the nature of repression.

Axmacher's response to the Argument Three—that psychoanalytic explanations are unfalsifiable and different in kind from scientific explanations—is to deny the transparency and unfalsifiablity of psychological explanation.

It has been suggested that these characteristics of hypotheses on causal relationships (to be surprising and falsifiable) do not apply for explanations of actions by a reason. According to this line of argumentation, a typical explanation from a first-person-perspective (e.g., “Why have you left the party?” – “Because it's time for bed and I want to go home”) cannot be falsified: in these explanations, “[… ] there is no question of ‘giving one's evidence’ or of ‘making a mistake’” (Toulmin, 1948, p. 25; quoted in Axmacher, 2013, p. 3).

Axmacher, in response to Toulmin, claims that first-person explanations may be neither transparent nor unfalsifiable, and thus no different in kind from causal explanations—“the transparency of the causes of one's own behavior is only fictitious” (Axmacher, 2013, p. 3), because we may be wrong about our reasons for acting. If this were all that Argument Three were saying, then Axmacher would be correct to dismiss it. However, Toulmin does not seem to be making the argument attributed to him. In his 1948 paper, Toulmin distinguishes *psychoanalytic* explanations from ordinary *psychological* explanations of behavior. A correct psychoanalytic explanation must fulfill three criteria, one of which is that “the patient must come to recognize it as a natural … expression of his neurotic state of mind” (Toulmin, 1948, p. 28); the other two criteria are that the psychoanalytic explanation must be plausible to a third party familiar with the patient, and the explanation must invoke relevant facts about the patient's early life. Toulmin states that a patient “must come to recognize” an explanation as the expression of his “neurotic state of mind:” this emphasizes that such explanations are usually not initially transparent to the person, and they later replace the ordinary psychological explanations of his actions. So Toulmin, contrary to Axmacher's suggestion, *is* aware that psychoanalytic explanations may at times fail to be transparent to an agent, and that they are therefore also falsifiable.

Toulmin does, however, distinguish between psychoanalytic explanations and other kinds of causal explanations; he states that we have “grounds for regarding the techniques of psycho-analysis as potentially “rational,” in a way in which hypnotic suggestion, brain surgery and insulin treatment cannot be” (Toulmin, 1948, p. 28). Toulmin says that in psychoanalysis we appeal to a person's *reasons*, and these tell a different causal story from brain surgery. Axmacher would disagree with Toulmin here as well; for a fuller discussion of why I think that Toulmin is right about this, I shall proceed to Argument Two; I argue that Axmacher's objections to Argument Two can be countered by those who think that there *is* an important distinction to be made between psychoanalytic explanations on the one hand, and causal explanations in the natural sciences on the other.

Argument Two claims that “causal explanations state an inevitable and lawful relationship between cause and effect” while “hermeneutic explanations rely on deferred reconstructions” (Axmacher, 2013, p. 2). Axmacher argues, however, that psychoanalytic explanations *can* be understood as causal, and that there is thus no difference in kind between causal and hermeneutic explanations. In defense of this claim, Axmacher offers the following:

When I ask someone, “Why didn't you come to work yesterday?” and he responds “Because I was sick,” then his staying at home is considered as an inevitable effect of his disease … this criterion exactly describes a characteristic feature of psychoanalytic interpretation (Axmacher, 2013, p. 2).

Axmacher concludes that psychoanalytic interpretations must tell a causal story because, like causal explanations in the sciences, they “give sufficient, but not necessary explanations of psychopathological symptoms” (Axmacher, 2013, p. 3). This seems right: if psychoanalysis did not appeal to causation, it would have little explanatory and therapeutic value. But are the causal stories the same? Those of us who think not would argue, following the philosopher Donald Davidson, that a central difference between psychological explanations, and causal explanations in the sciences, is that the latter, but not the former, appeal to generalizable laws. Davidson argued that, although psychological events, like physical events, feature in causal relations, only the latter feature in causal laws. We can cite causes and effects of particular psychological events, but we can never cite the law under which these events are subsumed, because there are no psychological laws. The mental realm, according to Davidson, is anomalous. This is not the case for the physical sciences. The relation between a disease and its symptom, for example, is explained by citing a general law—if you contract the HI virus, you are likely, *ceteris paribus*, to develop AIDS. Such a law is not exceptionless, but we are able to spell out, at least in principle, the nature of the relevant *ceteris paribus* conditions. But, as Davidson noted, this is not possible in psychological explanations:

What prevents us from giving necessary and sufficient conditions for acting on a reason also prevents us from giving serious laws connecting reasons and actions. To see this, suppose we had the sufficient conditions. Then we could say: whenever a man has such-and-such beliefs and desires, and such-and-such further conditions are satisfied, he will act in such-and-such a way. There are no serious laws of this kind (Davidson, 2001, p. 233).

There are no serious laws of this kind because we must always, as a matter of principle, refer back to the beliefs and desires of the agent: for instance, we may say that a person who believes that it is raining and who desires not to get wet will, *ceteris paribus*, take an umbrella outside. But we cannot spell out the nature of these *ceteris paribus* conditions: a person may not believe that the umbrella will keep him dry; or he may have a stronger desire to win a bet, or he may wish to get wet while singing in the rain. The set of disjunctives is open-ended as a matter of principle, whereas this is not the case in the physical sciences.

Davidson does not deny that we are able to cite sufficient conditions for a *particular* action, for he does not deny that psychological events are caused—by other psychological events, and also by physical events. A psychoanalyst can confidently predict that her paranoid analysand will become angry when she announces that she is taking a holiday because he has difficulties dealing with loss. But Davidson argues that these particular causal relations are not subsumable under general psychological laws: “Causality and identity are relations between individual events no matter how described. But laws are linguistic; and so events can instantiate laws, and hence be explained or predicted in the light of laws, only as those events are described in one or another way” (Davidson, 2001, p. 215). And events described as psychological cannot be explained or predicted on the basis of psychological laws because there are no such laws.

The philosopher Richard Wollheim makes a similar argument in discussing the central role that phenomenology plays in psychoanalysis. He writes:

“Cause” is a fully extensional relation, “causally explain” is not. If physics or physiology discovers that one event causes another, then we can assert this connection under any description of the two events … The same does not hold for “causally explains.” To say that one event causally explains another is true only for those descriptions of the two events which pick out whatever feature of them it is in virtue of which one causes the other (Wollheim, 1993, p. 89).

Thus, although both psychological and physical events feature in causal explanations, there is a fundamental difference between hermeneutic-type explanations characteristic of psychoanalysis (and folk psychology more broadly), and causal explanations characteristic of the sciences. This difference is a principled one—according to Davidson, the physical is subject to descriptive physical laws, while the psychological is normative, subject to the standards of rationality, constituted by mental holism, and anomalous. The absence of strict laws does not preclude the existence of psychoanalytic *generalizations* about human behavior, but these generalizations—unlike those of the natural sciences—are not expressible as strict causal laws. We cannot give an account of psychoanalytic behavior by appealing to the causal laws of the natural sciences without changing the subject.

If this is so, then, even if mental events feature in causal explanations, this is insufficient to argue for an integration of psychoanalysis with neuroscience. Axmacher's position does not get to the heart of the divide between psychoanalytic and scientific (including neuroscientific) explanations—namely that causal explanation in psychoanalysis describe phenomena in terms of radically different systems of concepts from those described in the neurosciences. Causal explanations in psychoanalysis make use of a fundamentally different system of kinds than those in the neurosciences. These fundamental differences raises serious questions about attempts to bring these two disciplines together, and should serve as a cautionary tale for both psychoanalytic metapsychology, and particularly for defenders of neuropsychoanalysis.

## Conflict of interest statement

The author declares that the research was conducted in the absence of any commercial or financial relationships that could be construed as a potential conflict of interest.
